# Are we over-conserving charismatic species?

**DOI:** 10.1371/journal.pbio.3003494

**Published:** 2025-12-03

**Authors:** Hai-Tao Shi, Yang Liu, Tien Ming Lee

**Affiliations:** 1 Ministry of Education Key Laboratory for Ecology of Tropical Islands, College of Life Science, Hainan Normal University, Haikou, China; 2 State Key Lab of Biological Control, School of Ecology, Sun Yat-Sen University, Shenzhen, China

## Abstract

Is all conservation good? This Perspective argues that the prevalent strategy of focusing on charismatic species may be counterproductive if conservation impact measures are oversimplistic and do not facilitate the restoration of long-term ecosystems and their functions.

## Teaser

The prevalent strategy of conserving nonmegafauna charismatic species may be counterproductive, if conservation impact measures are oversimplistic and do not facilitate the restoration of long-term ecosystems and their functions.

## Introduction

A balanced and healthy ecosystem requires the diversity and stability of many species that co-exist [1]. However, the local extinction of keystone, flagship, indicator, umbrella, and charismatic species may lead to cascading and profound ecological and social effects on the tangled web of species in ecosystems [2,3]. Such species may be repopulated and reintroduced through ecological interventions, although some species will grow unchecked, and often in excess [4]. This unintended consequence may be caused and reinforced by humans, sometimes under the guise of ecological preservation, with significant resources allocated.

The effectiveness of conservation is often evaluated by assessing biotic factors, such as key indicator species, and abiotic factors, such as soil and water conservation outcomes. When sound protection is offered, the number and quality of key indicator species will increase. However, the key indicator species is usually a community of species, and solely judging on the increase in the number of certain species may not necessarily point to conservation effectiveness. On the contrary, a more appropriate outcome reflects an ecosystem state that is constantly improving and ecologically balanced, characterized by rising biodiversity, improvement in the quality of ecological functions of the ecosystem, and increasing ecosystem stability [5]. Hence, improving and sustaining a balanced set of key indicator species and other related measures should be crucial for attaining a healthy and stable ecological environment, thereby avoiding the flagship species conservation conundrum [6].

Worldwide, there has been a focus on charismatic species conservation, with many successful attempts. For example, the Florida panther (*Puma concolor coryi*), Iberian lynx (*Lynx pardinus*), Yellowstone wolf (*Canis lupus*), and giant panda (*Ailuropoda melanoleuca*) are poster children of charismatic megafauna conservation, where wild and reintroduced populations have successfully rebounded after conservation intervention programs.

In recent years, China has dedicated extensive resources to conserving many key species (not just megafauna), at almost any cost. Unfortunately, many practitioners appear to unequivocally equate a large quantity of a charismatic species with a high-quality ecosystem. This oversimplification of conservation impact by considering a single outcome measure (i.e., maximizing the population of a charismatic species) has caused well-known problems for species such as the Chinese giant salamander (*Andrias davidianus*), crested ibis (*Nipponia nippon*), and Père David's deer (*Elaphurus davidianus*), to name just a few (see Box 1 for details). We need to tackle this entrenched problem of “overzealous” conservation in China and beyond.

**Box 1 Key examples of “over-conservation” of charismatic species in China.**

**Chinese giant salamander**

The Chinese giant salamander (*Andrias davidianus* sensu lato) is critically endangered owing to overexploitation. Wild populations were captured for domestication and artificial breeding, with individuals that were successfully raised released into the wild to demonstrate that in situ breeding can solve the problem of conserving wild populations of threatened species [7]. However, it was later discovered that this salamander, originally thought to be just one species, contained at least seven cryptic species [8]. As such, mixing a population admixture from multiple places and then releasing them everywhere was not only inappropriate for ecological preservation, but also diluted the natural and wild populations leading to genetic pollution.

**Crested ibis**

The number of crested ibis has increased from seven individuals in 1981 to more than 11,000 at present, an increase of a thousand-fold. The habitat area has also expanded from less than 5 to 16,000 square kilometers. Even though the IUCN Red List threat level of the crested ibis was downgraded from critically endangered to endangered in 2000, its population has grown in recent years due to in situ and ex situ protection and reintroduction. At least eight breeding centers focusing on its conservation have been established in Zhejiang, Sichuan, Shaanxi, Hebei, and other provinces throughout China, and the species has been reintroduced to many other locations in East Asia including Japan and South Korea [9]. Yet, this species has experienced problems such as highly dense populations in local areas, inbreeding depression, and increased mortality [10]. As enthusiasm for conservation research for this species continues to increase, these ongoing problems will worsen. Based on current niche models, habitats of medium and high suitability for crested ibises in China are estimated to be around 106,000 square kilometers. Under future climate change scenarios, the suitable habitat area for the species is predicted to increase by 1.4 million square kilometers, accounting for nearly a fifth of the country’s land area [11]. By solely focusing on the unlimited growth of a single protected species, we may neglect the importance of maintaining an ecologically balanced ecosystem. Notably, however, there is evidence of social and economic impacts from Shaanxi Province where, in order to protect the habitat for crested ibis, conventional agricultural practice had to be changed to organic farming of black rice (to reduce the use of pesticides and fertilizers).

**Père David's deer**

The Père David's deer became extinct in mainland China at the end of the 19th century due to habitat loss, climate change, and overhunting. In 1986, the Chinese government introduced 39 animals to the Dafeng Père David's deer National Nature Reserve in Jiangsu Province. After more than 30 years of artificial breeding and reintroduction into the wild, the artificially bred deer population has now returned to its original habitat. The number of ex situ protected areas has also increased from the original two to 89, supporting a population of more than 12,000 individuals, among which there are six wild populations, with one consisting of more than 5,000 deer; clearly a conservation success story [12]. However, reports have suggested that Père David’s deer are approaching the upper limit of their carrying capacity in some areas. As such, in recent years, there has been a growing call to artificially create microhabitats suitable for the survival of the hyperabundant deer to alleviate the pressure on existing habitats, as well as to explore new reserves on the beaches on both sides of the middle and lower reaches of the Yangtze River [13].

Over-conservation using simplistic outcomes has led to several issues (**Box 1**), including the mixing of population admixtures of cryptic species from captive breeding programs, which can lead to genetic pollution [7,8]. The unlimited growth of a single protected species can cause problems such as high densities of populations, inbreeding depression, and increased mortality, as well as a growing need for more suitable habitat [10,11]. In addition, an artificially bred population can approach the upper limit of their carrying capacity in certain areas, necessitating the creation of artificial microhabitats suitable for the survival of the hyperabundant species [13]. In addition to the cases discussed in Box 1, there have been many other similar misguided conservation programs, where the blind pursuit of increasing populations of charismatic species as the main and only goal overshadowed scientific species conservation plans. Notably, domestication and artificial breeding do not necessarily lead to the recovery of wild populations, as we have already seen with the Saiga antelope (*Saiga tatarica*), Chinese alligator (*Alligator sinensis*), wild horse (*Equus ferus*), and sika deer (*Cervus nippon*), to name just a few.

Tackling the entrenched problem of maximizing population requires practitioners and authorities to grasp that a balanced ecological environment is shaped by the long-term adaptation of a complex system of multiple biotic and abiotic factors. As such, we should expect to have a dynamic and adaptive set of conservation goals, particularly in light of global change. For example, due to climate change, protected areas must account for alterations in the climate niche envelope as part of species conservation outcomes [14]. Although the goals of decision makers may be commendable, the effectiveness of ecological restoration should not be only measured by species abundance. Many instances are not backed by science, where the underlying concepts and approaches are often flawed. If this trend persists, they will significantly influence future conservation undertakings to adopt similar practices. Instead, we argue that the more appropriate approach is to allocate limited resources for the protection of the ecological environment and to mitigate excessive human interventions.

There is a seeming misunderstanding regarding the relationship between the integrity of the environment and charismatic species. Balancing an ecosystem is a relatively dynamic process. When a species becomes extinct or experiences a significant decline in population, a new equilibrium will be established through natural adaptation. Whether to reintroduce the charismatic species or not would then have to be backed by new evidence. Reintroduction should be prioritized in regions where the species formerly thrived but is now extinct, but high numbers of a single charismatic species in the original ecosystem should be avoided. One reason why this simple and misleading approach is quite prevalent in China is that there is relatively poor consensus as to what a comprehensive evaluation entails. In the absence of a scientific assessment of a population target outcome, most practitioners are likely to continue rely on an increasing population as the primary indicator of success for securing additional funding for subsequent conservation efforts, slipping into a positive feedback loop and exacerbating the over-conservation issue.

In a nutshell, the present measures used to evaluate conservation impacts are not ecologically meaningful or too simplistic at best. In fact, we would argue that the issue of flagship-focused conservation is a bit outdated, as recent conservation initiatives for biodiversity protection are increasingly emphasizing functional aspects and whole-ecosystem approaches. Current progress has been inconsistent with the scientific theory and practice of holistic ecological protection. It not only fails to restore long-term ecosystems and their functions in accordance with nature’s laws but also results in the ineffective use of resources for biodiversity conservation. Yet, it remains a challenge for conservation strategy to be adaptive and keep pace with the dramatic and rapid alterations of the environment, particularly when it keeps deteriorating at unprecedented rates.

Insights from China should inform future efforts worldwide. We would urge all stakeholders to consider the issue of excessive conservation of charismatic species and emphasize that conservation efforts must be grounded in the scientific concept of a conservation goal to maintain overall ecological equilibrium as practically and realistically as possible.
